# Prostate-Specific Membrane Antigen in Anaplastic and Poorly Differentiated Thyroid Cancer—A New Diagnostic and Therapeutic Target?

**DOI:** 10.3390/cancers13225688

**Published:** 2021-11-14

**Authors:** Sabine Wächter, Pietro Di Fazio, Elisabeth Maurer, Jerena Manoharan, Corinna Keber, Andreas Pfestroff, Damiano Librizzi, Detlef K. Bartsch, Markus Luster, Friederike Eilsberger

**Affiliations:** 1 Department of Visceral, Thoracic and Vascular Surgery, University Hospital Marburg, 35043 Marburg, Germany; Pietro.Di-Fazio@med.uni-marburg.de (P.D.F.); Elisabeth.Maurer@med.uni-marburg.de (E.M.); Jerena.Manoharan@uk-gm.de (J.M.); Detlef.Bartsch@med.uni-marburg.de (D.K.B.); 2 Department of Pathology, University Hospital Marburg, 35043 Marburg, Germany; Corinna.Brehm@med.uni-marburg.de; 3 Department of Nuclear Medicine, University Hospital Marburg, 35043 Marburg, Germany; Andreas.Pfestroff@med.uni-marburg.de (A.P.); Damiano.Librizzi@med.uni-marburg.de (D.L.); markus.luster@med.uni-marburg.de (M.L.); friederike.mueller@staff.uni-marburg.de (F.E.)

**Keywords:** anaplastic thyroid carcinoma, poorly differentiated thyroid carcinoma, prostate-specific membrane antigen, theranostic, neovasculature, immunohistochemistry, treatment

## Abstract

**Simple Summary:**

Prostate-specific membrane antigen (PSMA)-targeted therapy represents a promising therapeutic option for the treatment of advanced carcinoma, but data on the relevance of PSMA-targeted diagnostics and therapy for anaplastic (ATC) and poorly differentiated (PDTC) thyroid carcinoma are still lacking. Due to the limited therapeutic options for these entities, the aim of this study was to evaluate to what extent Gallium-68 (^68^Ga)-PSMA-positron emission tomography/computed tomography (PET/CT) is superior to F-18-Fluordeoxyglucose (^18^F-FDG)-PET/CT in the diagnostic of ATC/PDTC and could represent a new therapeutic option in terms of theranostic. The findings of this study confirm the high diagnostic sensitivity and superiority of ^18^F-FDG-PET/CT in comparison to ^68^Ga-PSMA-PET/CT in the diagnosis of ATC and PDTC. However, it can be suggested that ^68^Ga-PMSA-PET/CT can be considered as a beneficial adjunct to the well-established ^18^F-FDG-PET/CT for a few individual selected patients with ATC and PDTC to detect lesions not discovered by ^18^F-FDG-PET/CT and to determine patients’ eligibility for a radioligand therapy. With regard to our results, radiolabelled PSMA-ligands with Lutetium-177 (^177^Lu)-PSMA may, in the future, represent a theranostic approach with only minor side effects for a few individual selected patients with ATC and PDTC who need alternative treatment options in case of progression when established therapies are no longer effective.

**Abstract:**

Several studies have demonstrated an expression of the prostate-specific membrane antigen (PSMA) in the cancer-related neovasculature of thyroid malignancies. Due to the poor prognosis and limited therapeutic options for patients with anaplastic (ATC) and poorly differentiated (PDTC) thyroid carcinoma, the aim of our study was to investigate the theranostic approach of PSMA expression in these patients. The PSMA uptake on Gallium-68 (^68^Ga)-PSMA-positron emission tomography/computed tomography (PET/CT) and glucose uptake on F-18-Fluordeoxyglucose (^18^F-FDG)-PET/CTs were analysed in two ATC and six PDTC patients. The PSMA expression in corresponding patients’ tissue samples was detected by immunohistochemistry. In addition, various tissue sections from 22 ATC and six PDTC patients were examined concerning PSMA expression. ^68^Ga-PSMA-PET/CT showed heterogeneous PSMA expression among patients and lesions. Six of the eight analyzed patients (two ATC, four PDTC) showed increased glucose metabolism without increased PSMA uptake after PET/CT. In one patient (PDTC), ^18^F-FDG-PET/CT tracer uptake was positive and ^68^Ga-PSMA-PET/CT showed heterogeneous results. Another patient (PDTC) evidenced only PSMA-positive lesions and received two cycles of Lutetium-177 (^177^Lu)-PSMA therapy, which kept his disease stable for seven months. There was a correlation between immunohistochemical PSMA expression and uptake on ^68^Ga-PMSA-PET/CT in three of the examined patients. Twenty-seven of the analyzed 39 ATC and 13 of the analyzed 22 PDTC tissue sections showed a strong PSMA expression. Considering the rarity of PDTC and ATC, which is the reason for the small patient population we studied, the findings of this study confirm the high diagnostic sensitivity and superiority of ^18^F-FDG-PET/CT in comparison to ^68^Ga-PSMA-PET/CT in the diagnosis of ATC and PDTC. However, it can be suggested that ^68^Ga-PMSA-PET/CT can be considered as a beneficial adjunct to the well-established ^18^F-FDG-PET/CT for a few individual selected patients with ATC and PDTC to detect lesions not discovered by ^18^F-FDG-PET/CT and to determine patients’ eligibility for a radioligand therapy. Radiolabelled PSMA-ligands may, in the future, represent a theranostic approach with only minor side effects for a few individual selected patients with ATC and PDTC who need alternative treatment options in case of progression when established therapies are no longer effective. However, due to the small sample size of our collective, larger studies are needed to allow for a final evaluation on the significance of PSMA-targeted diagnostic and therapy for ATC and PDTC.

## 1. Introduction

Anaplastic (ATC) and poorly differentiated thyroid carcinoma (PDTC) represent less than 5% of thyroid cancers [1,2]. However, they are clinically relevant entities because of their extremely poor prognosis depending on their aggressive behaviour and high metastatic potential. ATC accounts only for approximately 1–2% of thyroid cancer. Patients affected by ATC have a mean survival time of 3–6 months and a mortality rate exceeding 90% [3,4,5,6,7]. The prognosis of PDTC, whose incidence varies between 1–15% is slightly better with a median 5-year-survival of 62–85%; nevertheless, PDTC is responsible for the majority of all fatalities from non-anaplastic follicular cell derived thyroid cancer [8,9,10,11]. Despite multimodal treatment strategies based on surgical resection in combination with radiochemotherapy and novel targeted therapies, including multikinase inhibitors (mKI), the prognosis of ATC and PDTC could not be significantly improved over the last 20 years [1,7,11,12]. New options to develop innovative diagnostic and therapeutic approaches for the treatment of these aggressive carcinomas have arisen in the field of theranostics and, in particular, by detecting prostate-specific membrane antigen (PSMA) [1,5,13,14,15,16]. Theranostics using radiolabelled PSMA-ligands have gained significant influence in the management of prostate cancer. Meanwhile, targeted radionuclide therapy—for example, with Lutetium-177 (^177^Lu) labelled PSMA peptides—represents a promising treatment option for castration-resistant prostate cancer, as currently confirmed in the VISION study (ClinicalTrials.gov Identifier: NCT03511664) [17,18]. Increased PSMA expression has also been observed in other carcinomas [19,20]. Interestingly, the over-expression of PSMA was histopathologically detected in the neovasculature of PDTC and ATC. Some case reports have already shown a high uptake on Gallium-68 (^68^Ga)-PSMA-positron emission tomography/computed tomography (PET/CT) in the primary tumor, cervical and mediastinal lymph nodes in ATC. However, the value of theranostic concepts using radiolabelled PSMA ligands for the diagnostic and treatment of ATC and PDTC remains still unclear [21].

Therefore, the aim of this study was to investigate the expression of PSMA in tissues from patients suffering from ATC and PDTC and to correlate the extent of expression with the feasibility and utility of the imaging with ^68^Ga-PSMA-PET/CT. In particular, we evaluated to what extent ^68^Ga-PSMA-PET/CT is superior to F-18-Fluordeoxyglucose (^18^F-FDG)-PET/CT in the diagnostic of ATC/PDTC and could represent a new therapeutic option in terms of theranostics for these carcinomas.

## 2. Materials and Methods

### 2.1. Patient Selection

This is a single-center retrospective pilot study approved by Marburg University Hospital ethics committee (Az. 27/21), which included two patients with histologically confirmed ATC and six patients with PDTC treated between 2007 and 2021 at the Department of Visceral, Thoracic and Vascular Surgery and the Department of Nuclear Medicine at the Philipps University Hospital of Marburg. All of the patients enrolled in this study received a ^68^Ga-PSMA-PET/CT in addition to ^18^F-FDG-PET/CT.

Data on patients’ characteristics, including gender, age, treatment history, histopathological findings and information on diagnostic procedures involving ^68^Ga-PSMA-PET/CT, in addition to ^18^F-FDG-PET/CT, were retrieved from the medical files. Corresponding to the clinical data and PET/CT results, the PSMA expression in immunohistochemistry (IHC) was detected in paraffin-embedded formalin-fixed tissue sections from primary tumors, lymph nodes, pulmonary, soft tissue and osseous metastases, as well as from local tumor recurrences from these patients. We included one patient with PDTC for PET/CT evaluation in whom no tissue could be examined.

### 2.2. Imaging Protocol and Analyses of ^18^F-FDG- and ^68^Ga-PSMA-PET/CT

Both ^18^F-FDG-PET/CT and ^68^Ga-PMSA-PET/CT were performed according to the recommendations of the German Society of Nuclear Medicine [22].

When ^18^F-FDG-PET/CT was performed, a median activity of 265.5 MBq (range 248–297 MBq) Fluorine-18-FDG was applied. The median blood glucose value at the time of application was 99.5 mg/dL (range 73–122 mg/dL). After a median time of 60 min after application, image acquisition was performed, which was contrast media-enhanced in five patients. 

^68^Ga-PMSA-PET/CT was performed after application of a median activity of 168 MBq (range 140–212 MBq) Gallium-68-PSMA after a median time of 60 min. This examination was performed with contrast agent in three patients.

An experienced nuclear medicine physician evaluated the PET/CTs. The experienced physician considered a lesion positive, after visually sufficient increased tracer accumulation.

### 2.3. Immunohistochemical Analysis

#### 2.3.1. Tissue Selection

In addition to the tissue samples from the seven patients who received a ^68^Ga-PSMA-PET/CT, PSMA expression was further detected in tissue sections from 22 ATC patients and six PDTC patients that have previously undergone surgery or diagnostic punctures at the Department of Visceral, Thoracic and Vascular Surgery or the Department of Nuclear Medicine at the Philipps University Hospital of Marburg.

Overall, the histopathological analyses included 22 ATC primary tumors, five ATC lymph node metastases, four pulmonary/pleural metastases of ATC, seven metastases of the soft tissue surrounding the thyroid gland and one ATC presternal metastasis. Furthermore, six PDTC primary tumors, six PDTC lymph node metastases, three pulmonary metastases, one renal metastasis, two local recurrences and four osseous metastases of PDTC were evaluated with regard to their PSMA expression. 

#### 2.3.2. Immunohistochemical Staining for PSMA Antigens

Immunohistochemistry (IHC) was performed on 1,2 µm-thick paraffin-embedded formalin-fixed tissue sections. Sections were deparaffinised in xylene and subsequently rehydrated through ethanol. Heat-induced epitope retrieval was performed with trilogy. Staining was performed on a DAKO auto-stainer plus. After blocking of endogene peroxidase, sections were incubated for 45 min with mouse monoclonal anti-PSMA antibody (1:4000; ABIN 1302364, clone GCP-04; antibodies-online GmbH, Aachen, Germany). Sections were washed and incubated with Dako REAL EnVision HRP Rabbit/Mouse polymer (Agilent Technologies Deutschland GmbH, Waldbronn, Germany), which reacts with DAB-Chromogen, according to the manufacturer´s protocol.

#### 2.3.3. Assessment of PSMA Expression

Assessment of PSMA expression was calculated with the method described by Heitkötter et al. [23,24] and evaluated by an experienced pathologist (CK) on immune-stained whole ATC and PDTC slides. PSMA expression in either tumor cells or tumor-associated vessels was considered positive. Staining intensity was scored semiquantitatively as negative (0), weak (1 = barely perceptible staining at higher (400×) magnification), moderate (2 = readily apparent at lower power (40×) magnification) or strong (3). The fraction of PSMA positive cells was assessed as <5% or >5%. In the case of heterogeneous staining, the predominant pattern was recorded. A weak (1) or moderate (2) staining intensity in <5% of the neovasculature and a weak (1) staining intensity in >5% of the neovasculature was allocated to the “low expression” group (PSMA-labelling index = 1), whereas a moderate (2) staining intensity in >5% of the neovasculature and a strong (3) staining intensity in < or > 5% of the neovasculature was assigned to the “strong expression” group (PSMA-labelling index = 2). In contrast to Heitkötter et al., we also implemented the PSMA-labelling index = 3, which classified a strong (3) staining intensity not only in the neovasculature, but also in the tumor cells themselves.

## 3. Results

### 3.1. Patient Characteristics

Two male patients with ATC and six patients with PDTC (four male, two female) underwent a ^68^Ga-PSMA-PET/CT in addition to a ^18^F-FDG-PET/CT.

The median age of the patients suffering from PDTC was 60 years (range 44–70 years) at initial diagnosis. All patients developed distant metastatic disease: (a) pulmonary metastases in three PDTC patients over a median time span of 13 months (range 0–56 months); (b) osseous metastases in four patients with a median occurrence after initial diagnosis of 7.5 months (range 0–41 months); (c) cerebral metastasis in one patient 44 months after the first diagnosis, respectively. This patient also had evidence of inguinal lymph node metastasis and soft tissue metastasis of the thoracic wall 41 months after the first diagnosis (patient 7).

Furthermore, we evaluated a 59- and 47-year-old patient with ATC (patient 1 and 2). One of these patients already had an advanced Stage IVC (Union internationale contre le cancer (UICC) (Brierley J, Gospodarowicz MK (Mary K., Wittekind C (Christian). TNM classification of malignant tumours. 253 p.) carcinoma with evidence of lymph node and pulmonary metastases at the time of initial diagnosis (patient 2). In the other ATC patient, the presence of pulmonary metastases was detected eight months after initial diagnosis (patient 1). Patients´ characteristics and previous treatments are shown in Table 1.

### 3.2. ^68^Ga-PSMA-PET/CT

The overall median time span between initial diagnosis and the prior PET of the particular patient examined in this study was 20.5 months (range 5–112 months)—for ATC seven and eight months and for PDTC 33.5 months (range 5–112 months).

In each case, we evaluated the ^18^F-FDG-PET/CT with the shortest time interval to the performed ^68^Ga-PSMA-PET/CT. In seven patients, a ^18^F-FDG-PET/CT was performed previously and the interval time between this prior ^18^F-FDG-PET/CT and ^68^Ga-PSMA-PET/CT in temporal connection was median 76 days (range 1–232 days). Only one of the evaluated PDTC patients (patient 6) received a ^68^Ga-PSMA-PET/CT first and subsequently underwent further imaging via ^18^F-FDG-PET/CT 171 days later. ^68^Ga-PSMA-PET/CT was performed in this patient in the setting of theranostics when therapeutic options were exhausted after two different mKI (sorafenib and lenvatinib) and re-challenge of one mKI (sorafenib) (patient 6).

Therefore, the overall evaluation time interval between ^18^F-FDG-PET/CT and ^68^Ga-PSMA-PET/CT amounted to a median 87.5 days (range 1–232 days). These data are included in the Table 2.

### 3.3. Case Series

Based on the examined patients, the following three possibilities have been identified:

1. Patients, in whom ^18^F-FDG-PET/CT uptake was positive and ^68^Ga-PSMA-PET/CT negative. This was the case in six of the eight patients evaluated (patient 1–6).
2. Patients, in whom ^18^F-FDG-PET/CT uptake was positive and ^68^Ga-PSMA-PET/CT showed heterogeneous results. This was the case in one of the eight patients evaluated (patient 7).
3. Patients, in whom ^68^Ga-PSMA-PET/CT uptake was clearly positive and thus a therapeutic option in terms of theranostics—for example, with a ^177^Lu-PSMA therapy—exists. This was the case in one of the eight patients evaluated (patient 8).

In the following, we will describe a detailed case example for each of the three possibilities mentioned above. These data can be found along with further information on each patient in Table 1 and Table 2. For a better overview we evaluated the five most conclusive metastases, if more than ten metastases of one site were present in a patient.

#### 3.3.1. Setting No. 1—^18^F-FDG-PET/CT Positive, ^68^Ga-PSMA-PET/CT Negative

##### Patient 1: Male, ATC, 59 Years Old at the Time of Diagnosis

As part of the follow-up after performing thyroid surgery, as well as radiochemotherapy and initiation of mKI therapy, ^1^^8^F-FDG-PET/CT and ^68^Ga-PMSA-PET/CT were performed seven months after initial diagnosis. The time interval between these two examinations was one day. 

**Figure 1:**
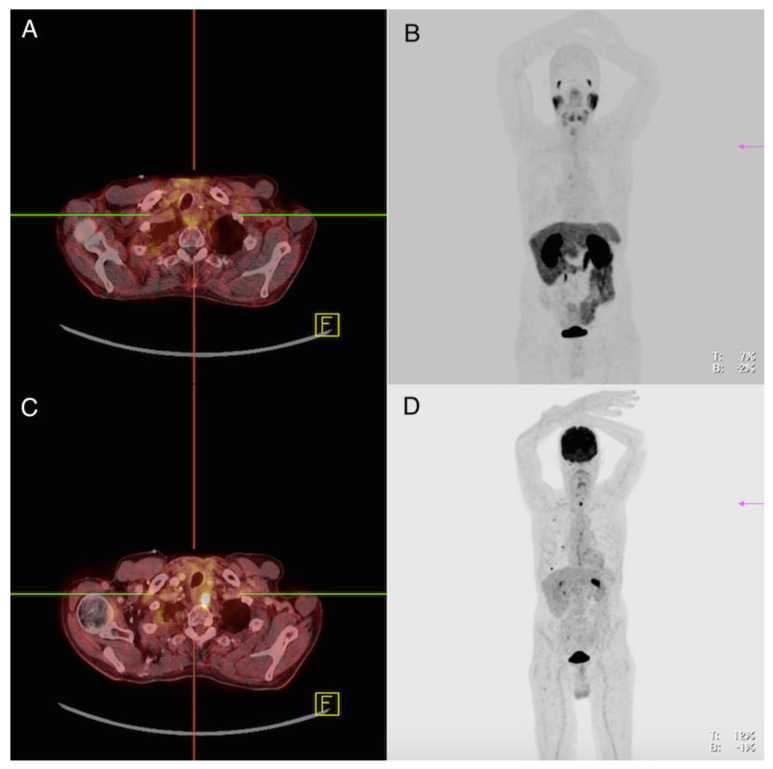
Patient 1. ^68^Ga-PSMA-PET/CT (**A**,**B**), ^18^FDG-PET/CT (**C**,**D**). The finding was compatible with a local recurrence in the left thyroid bed. The FDG-positive lesion showed no PSMA expression.

Imaging analysis: ^18^F-FDG-PET/CT showed in the area of the left thyroid bed near the esophagus a soft tissue lesion of 1.9 cm with increased glucose utilization (standardized uptake value (SUV)max 16.3); ^68^Ga-PMSA-PET/CT did not show pathologically increased tracer accumulation at this location (SUVmax 2.5) (Figure 1). Furthermore, ^18^F-FDG-PET/CT revealed one lymph node of 2.2 cm with slightly increased glucose accumulation in the mediastinum with suspicion of lymph node metastasis, which did not show clearly increased uptake on ^68^Ga-PMSA-PET/CT (SUVmax 3.3) and seven pleural lesions between 0.3–1.2 cm with increased glucose uptake (SUVmax 2.7 (range 1.5–7.9). None of these lesions showed increased PSMA uptake (SUVmax 1.3 (range 0.6–2.6).

**Figure 2:**
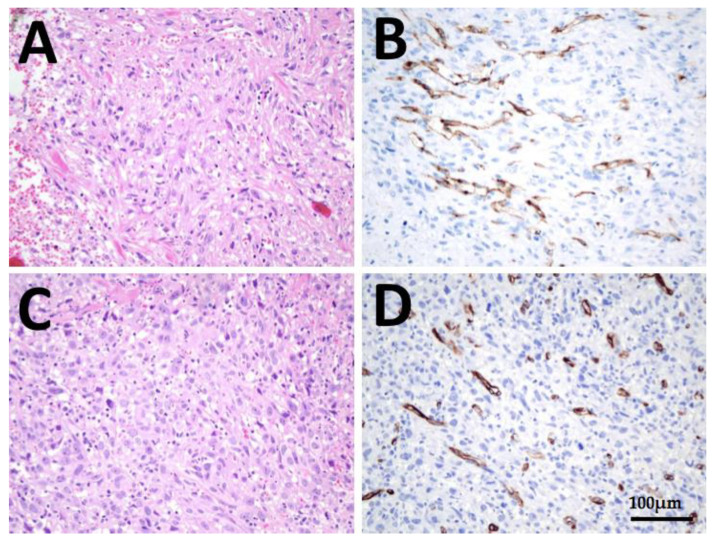
Immunohistochemistry of patient 1: (**A**,**B**), histology of ATC thyroidal primary tumor and (**C**,**D**) tumor infiltration of the soft tissue surrounding the thyroid gland in HE staining and PSMA immunohistochemistry, respectively. Strong immunoreactivity of more than 5% of neovasculature in the tumor can be seen at both localizations (PSMA labelling-index 2). Scale bar, 100 µm, respectively.

IHC analysis: The primary tumor as well as tumor infiltration of the soft tissue surrounding the thyroid gland could be pathologically investigated concerning the PSMA expression—both samples showed a “strong expression” (PSMA-labelling index = 2) (Figure 2).

In summary: This patient showed multiple metastases with increased glucose metabolism without increased PSMA uptake on PET/CT, despite a strong PSMA expression in the examined tissue samples.

Due to the fact that patient 2, 3, 4, 5 and 6, all suffering from ATC/PDTC, showed similar to patient 1 metastases of ATC/PDTC with increased glucose metabolism on ^18^F-FDG-PET/CT without an increased PSMA uptake on PET/CT, we provide further detailed information to these six patients in Table 1 and Table 2 as well as case descriptions including figures as supplementary material.

#### 3.3.2. Setting No. 2—^18^F-FDG-PET/CT Positive, ^68^Ga-PSMA-PET/CT Heterogenous

##### Patient 7: Female, PDTC, 61 Years Old at the Time of Diagnosis

After surgery of the thyroid gland in 2013, twofold radioiodine therapy in 2013, surgery of lymph node metastasis in 2014, a ^177^Lu -DOTATATE therapy in 2014, an mKI therapy with sorafenib in 2014 and external beam therapy of cervical vertebral and cervical lymph node metastases in 2016 ^18^F-FDG-PET/CT was performed 41 months after initial diagnosis. ^68^Ga-PMSA-PET/CT was conducted 76 days later. In the time interval between the two PET/CTs, this patient received the mKI lenvatinib, which she took for 52 days and then discontinued when she experienced angina pectoris symptoms.

**Figure 3:**
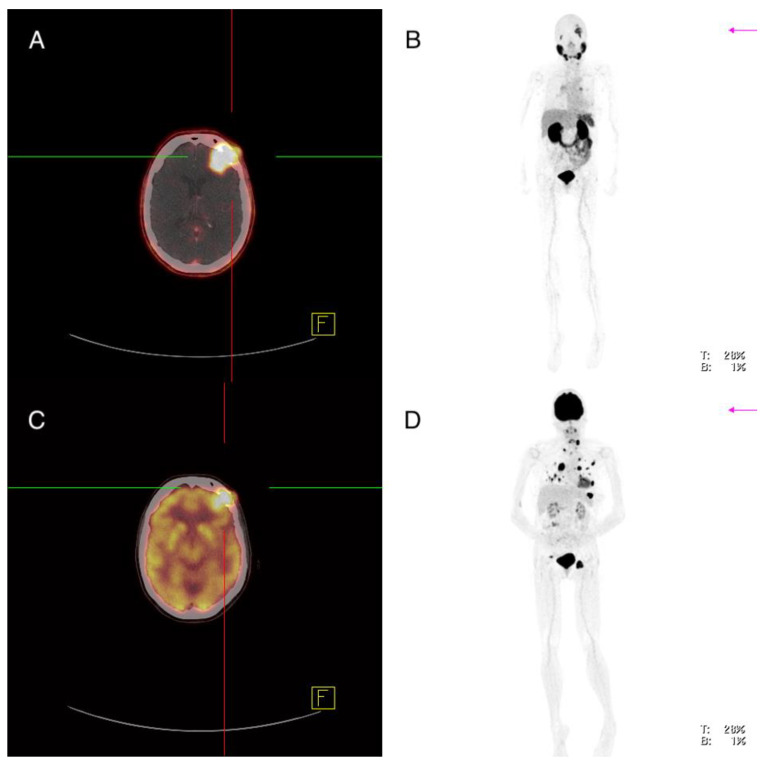
Patient 7: ^68^Ga-PSMA-PET/CT (**A**,**B**), ^18^FDG-PET/CT (**C**,**D**). A cerebral metastasis left frontal shows increased PSMA expression in concordance to an increased FDG uptake.

**Figure 4:**
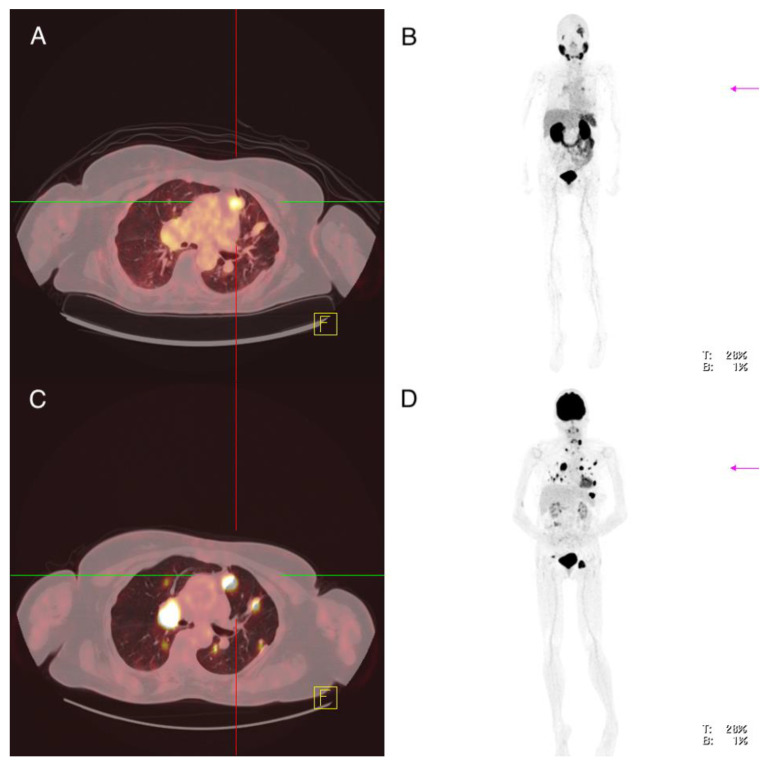
Patient 7: ^68^Ga-PSMA-PET/CT (**A**,**B**), ^18^FDG-PET/CT (**C**,**D**). Inhomogeneous PSMA expression of mediastinal lymph node metastases in the absence of PSMA expression of pulmonary lesions with increased glucose uptake.

Imaging analysis: ^18^F-FDG-PET/CT showed multiple metastases in various organs including a left frontal cerebral metastasis of 2.9 cm with increased glucose utilization (SUVmax 31.4), which also showed increased PSMA expression (SUVmax 12.3) (Figure 3). There were several lymph nodes with pathologically increased FDG uptake—for example, cervical on the left side of 2.0 cm, mediastinal in region 4 on the right of 3.6 cm and in region 5 of 1.4 cm and inguinal on the left of 5.4 cm (SUVmax 32.1; 36.4; 25.3; 35.2), with the lymph node in region 5 showing clearly increased PSMA expression (SUVmax 10.1) and the other lymph nodes showing discrete or partial PSMA expression (SUVmax 7.4; 6.3; 6.2) (Figure 4). A soft tissue metastasis of the thoracic wall of 2.1 cm was revealed after the detection of increased glucose metabolism; ^68^Ga-PMSA-PET/CT showed no increased PSMA expression (SUVmax 4.3). An FDG-positive osseous metastasis of the skull of 2.9 cm (SUVmax 16.9) showed discrete PSMA expression (SUVmax 7.2). Multiple pulmonary metastases showed an increased FDG uptake, for example, in the right lung two lesions in segment 10 of 1.1 and 1.8 cm (SUVmax 14.1; 12.5) and in the left lung segment 1 1.0 cm, segment 8 1.6 cm and segment 9 1.1 cm (SUVmax 13.3; 11.7; 11.7)—none of the pulmonary lesion showed increased PSMA expression (SUVmax 3.3; 4.0; 2.1; 3.5; 2.8) (Figure 4).

IHC analysis: The primary tumor, as well as a cervical lymph node metastasis, were examined with regard to their PSMA expression and revealed a “strong expression” (PSMA-labelling index = 2).

In summary: There was a heterogeneous PSMA expression of the metastases on PET/CT and a strong PSMA expression in the examined tissue samples.

#### 3.3.3. Setting No. 3—^68^Ga-PSMA-PET/CT Clearly Positive, Theranostic Approach

##### Patient 8: Male, PDTC, 59 Years Old at Time of Diagnosis

After thyroid surgery in 2011, the resection of a vertebral metastasis in 2015, cervical re-exploration with residual thyroidectomy without tumor detection in 2015 and radioiodine therapy in 2015, the patient received a ^18^F-FDG-PET/CT five months after initial diagnosis. 

**Figure 5:**
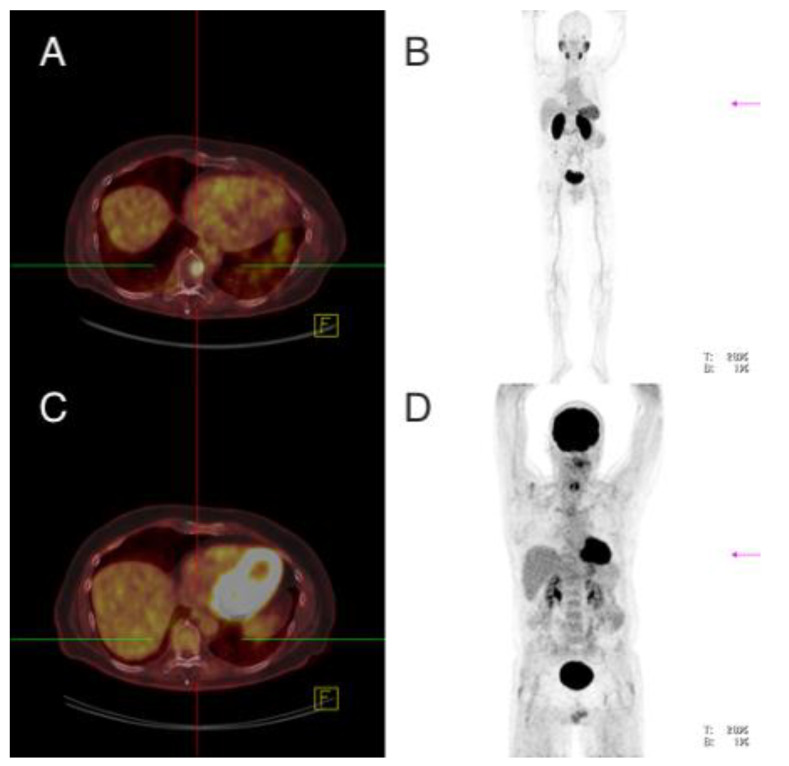
Patient 8. ^68^Ga-PSMA-PET/CT (**A**,**B**), ^18^FDG-PET/CT (**C**,**D**). Osseous metastasis thoracic vertebral body 9 with increased PSMA expression without increased glucose utilization.

**Figure 6:**
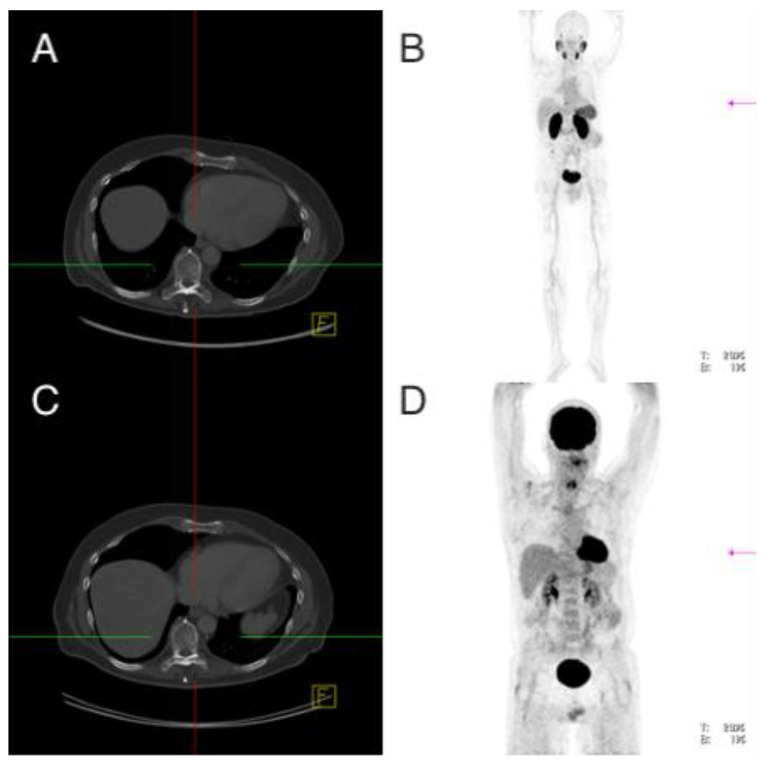
Patient 8. ^68^Ga-PSMA-PET/CT (**A**,**B**), ^18^FDG-PET/CT (**C**,**D**). Morphologic pattern of thoracic vertebral body 9 with osseous metastasis in CT.

Imaging analysis: While the first performed ^18^F-FDG-PET/CT showed no lesions with increased glucose metabolism, four osseous lesions with clearly (9th thoracic vertebra 1.6 cm, SUVmax 7.1; Os ilium right 1.2 cm, SUVmax 9.0) or discretely (7th rib without morphological correlate, SUVmax 2.9; acetabulum left 1.3 cm, SUVmax 4.0) pathologically increased PSMA expression (overall median SUVmax 5.55 (2.9–9.0)) were detected after the ^68^Ga-PSMA-PET/CT, which was performed 81 days later (Figure 5 and Figure 6).

**Figure 7:**
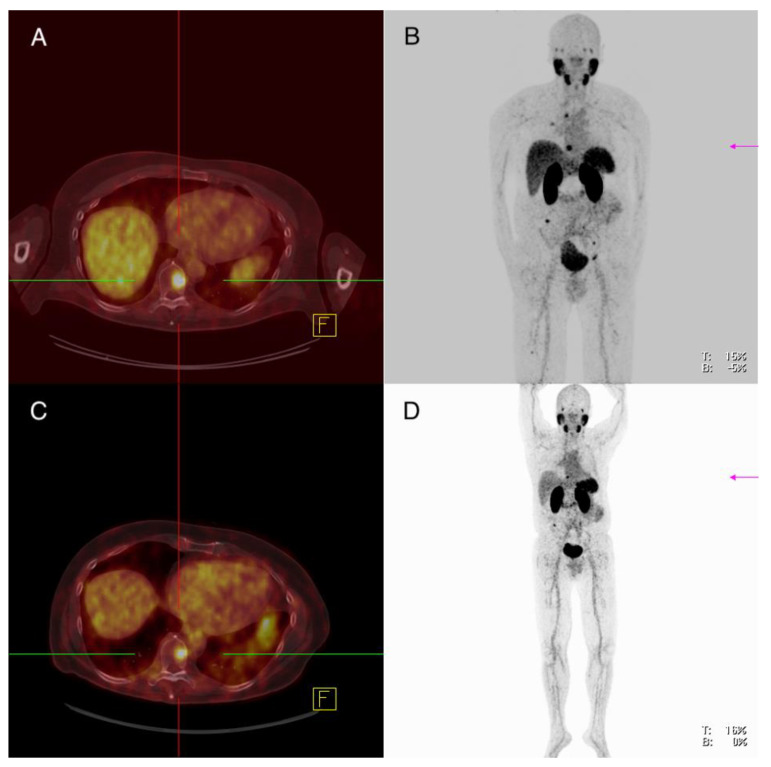
Patient 8. Second ^68^Ga-PMSA-PET/CT (**A**,**B**), first ^68^Ga-PMSA-PET/CT (**C**,**D**). Second ^68^Ga-PSMA-PET/CT: Progressive disease with size progression of known osseous lesions and multiple new osseous lesions with increased PSMA expression.

**Figure 8:**
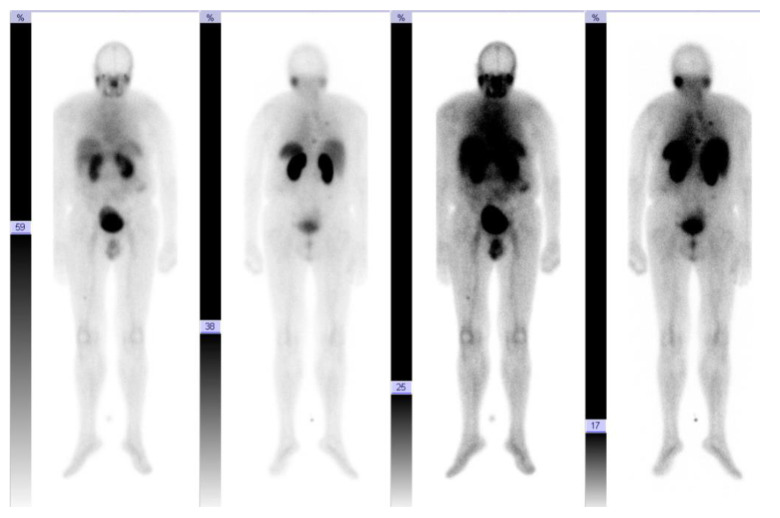
Patient 8. Whole-body-scan 90 min post first ^177^Lu-PSMA application with an intensive accumulation in the metastases.

**Figure 9:**
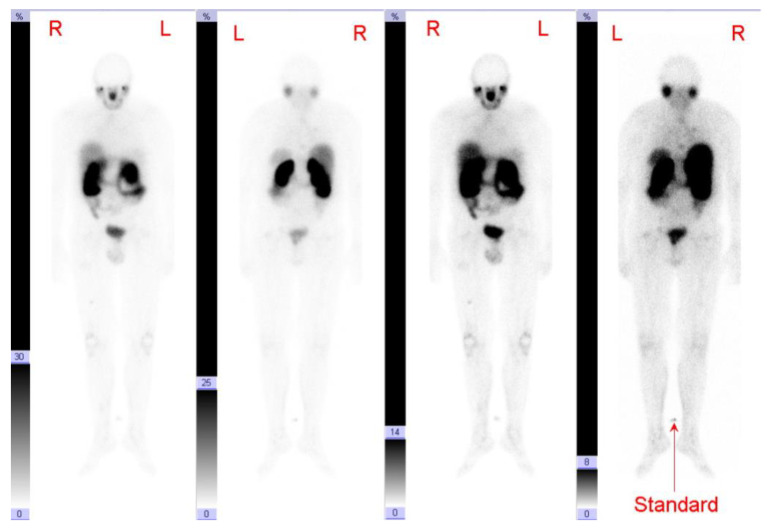
Patient 8. Whole-body-scan 43 h after the second ^177^Lu-PSMA application with an intensive nuclide accumulation in the metastases.

A second ^68^Ga-PSMA-PET/CT which was not included in our comparative analysis and which was conducted 187 days later, revealed a disease progression with multiple lesions with clearly pathologically increased PSMA expression (median SUVmax 10.0 (6.0–15.0) (Figure 7). Therefore, the patient received two cycles of ^177^Lu-PSMA therapy in the sense of theranostics within the framework on an individual therapeutic approach (Figure 8 and Figure 9). Based on the experience gained in the treatment of prostate and neuroendocrine carcinomas, 6.3 GBq were applied intravenously in the first cycle and 7.4 GBq were administered in the second cycle eight weeks later. Despite the fact that no side effects were evidenced after the first cycle, the patient experienced temporary nausea after the second one. During follow-up, stable disease was described until seven months after the first cycle, after which there was an increase in thyroglobulin levels; another three months later, FDG-positive metastases appeared for the first time in an ^18^F-FDG-PET/CT performed in the context of the tumor marker elevation.

IHC analysis: Unfortunately, no tissue sample could be examined for this patient.

### 3.4. Imaging Findings

Disease locations, median lesions SUVmax and the following analysis are listed in Table 3. Out of 11 evaluated ATC lesions, 10 were clearly visually FDG-positive (detection rate 91%) and none were clearly PSMA-positive (detection rate 0%). The detection rate for PDTC lesions was 89% for ^18^F-FDG-PET/CT (out of 37 evaluated PDTC lesions 33 were visually FDG-positive) and 11% for ^68^Ga-PMSA-PET/CT (out of 36 evaluated PDTC lesions four were visually PSMA-positive).

Discordant lesions in PDTC with explicit increased FDG uptake and no clear PSMA expression were seen in bone metastases (5/10), cervical lymph node metastases (1/2), mediastinal lymph node metastases (2/4), pulmonary metastases (15/15), local tumor recurrence/thyroid bed lesions (2/2) and soft tissue metastases (1/2); discordant lesions with explicit PSMA expression and no FDG uptake were seen in bone metastases (2/10) and concordant lesions with increased FDG uptake and increased PSMA expression were seen in a brain metastases (1/1) and a mediastinal lymph node metastasis (1/4). 

In ATC patients, discordant lesions with clearly increased FDG uptake and no clear PSMA expression were observed in pulmonary metastases (9/9) and local tumor recurrence/thyroid bed lesions (1/1). We observed no discordant lesion with explicit/increased PSMA expression and no or decreased FDG uptake in ATC.

Overall, median lesion SUVmax was 5.0 for ^18^F-FDG-PET/CT (range 1.5–28.3) and 1.5 for ^68^Ga-PMSA-PET/CT (range 0.6–4.0) in ATC and 17.9 for ^18^F-FDG-PET/CT (range 1.2–67.9) and 4.0 for ^68^Ga-PMSA-PET/CT (range 1.8–12.3) in PDTC, respectively. 

In ATC, SUVmax was lowest for mediastinal lymph node lesion (SUVmax 2.7) for ^18^F-FDG-PET/CT and for pulmonal lesions for ^68^Ga-PMSA-PET/CT (median SUVmax 1.3 (range 0.6–4.0)) and highest for tumor recurrence/thyroid bed lesions for ^18^F-FDG-PET/CT (SUVmax 16.3) and for mediastinal lymph node lesions for ^68^Ga-PMSA-PET/CT (median SUVmax 3.3).

In PDTC median SUVmax was lowest for osseous lesions (median SUVmax 9.9 (range 1.2–31.2)) for ^18^F-FDG-PET/CT and for local tumor recurrence/thyroid bed lesions for ^68^Ga-PMSA-PET/CT (median SUVmax 3.3 (range 2.4–4.2)); highest for tumor recurrence/thyroid bed lesions for ^18^F-FDG-PET/CT (SUVmax 43.5 (range 19.1–67.9)) and for cerebral metastases for ^68^Ga-PMSA-PET/CT (SUVmax 12.3).

### 3.5. Correlation between the Extent of Immunohistochemical PSMA Expression in Tissue Samples and Imaging in ^68^GA-PSMA-PET/CT with Regard to Different Metastatic Sides of ATC and PDTC Patients

In seven of the eight patients we examined, tissue samples of metastatic sides could be evaluated with regard to their PSMA expression and compared with the respective imaging results on ^68^Ga-PSMA-PET/CT (Table 4). In this regard, we only evaluated the metastases for which there was also a specific reproducibility in ^68^Ga-PSMA-PET/CT.

An increased PSMA expression in ATC or PDTC tissue samples and concordantly PSMA-positive lesions in ^68^Ga-PSMA-PET/CT was only observed in a cervical lymph node metastasis of a patient with PDTC (strong PSMA expression and discreet PSMA-positive lesion in PET/CT). In all other tissue samples examined, there was either a discordance between PSMA expression and uptake on ^68^Ga-PSMA-PET/CT or a low PSMA expression in IHC was associated with an absent uptake on PET/CT. In this regard, however, it must be noted that from patient 8 in particular, who evidenced clearly PSMA-positive lesions in PET/CT, no tissue samples were available and therefore could not be included in this investigation.

### 3.6. Immunohistochemical Analysis

#### PSMA Expression in Anaplastic and Poorly Differentiated Thyroid Cancer

PSMA expression was found in 36 of 39 analysed ATC and 21 of 22 PDTC tissue samples (Table 5) and was—with one exception—detected exclusively in the tumor microenvironment (tumor-associated neovasculature), but not in the tumor cells themselves.

27 of the analyzed ATC tissue sections showed a strong PSMA expression and nine displayed a weak expression. In 13 ATC patients, the PSMA expression was analyzed in the primary tumor as well as in existing metastases. Nine of these 13 patients displayed a concordantly high PSMA expression in the primary tumor as well as in the metastases; the PSMA expression was higher in the primary tumor than in the metastases in only four patients. In none of the analyzed ATC samples was the PSMA expression higher in the metastases than in the primary tumor.

13 of the analyzed PDTC tissue sections displayed a strong and six a weak PSMA expression in the tumor-associated neovasculature. From a total of six PDTC patients, the primary tumor and tissue sections of metastases were examined. In this context, PSMA expression in the primary tumor and associated metastases were consistently high in four of these patients. In one patient, a high PSMA expression was detected in the primary tumor and in the pulmonary metastasis, while the osseous metastasis showed no PSMA expression. In the other patient, PSMA expression was low in the primary tumor and high in the corresponding pulmonary metastasis.

**Figure 10:**
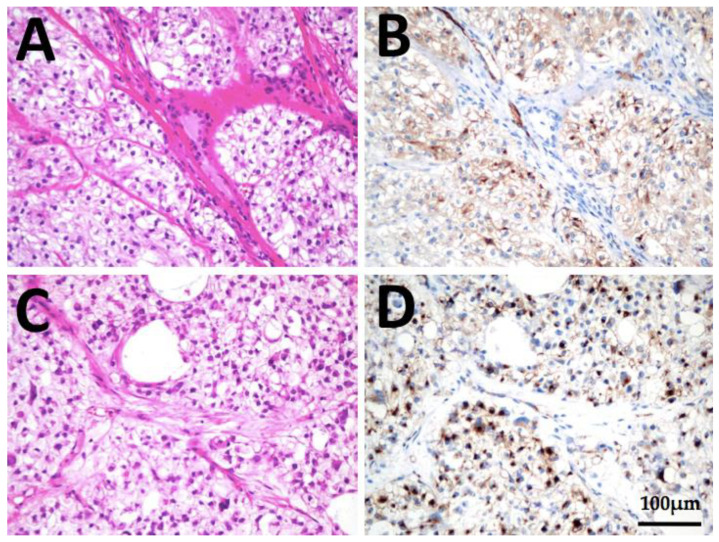
Immunohistochemistry of a patient with PDTC. (**A**,**B**), histology of PDTC thyroidal primary tumor and (**C**,**D**) cervical lymph node metastasis in HE staining and PSMA immunohistochemistry, respectively. Strong membraneous and cytoplasmatic immunoreactivity of tumor cells can be seen at both localizations (PSMA-labelling index 3). Scale bar, 100 µm, respectively.

One PDTC tumor sample showed an unusually high PSMA expression with a strong membraneous and cytoplasmatic immunoreactivity; not only of the neovasculature, but also of the tumor cells themselves, compared to all other analyzed tissue samples (Figure 10). Thus, we assigned a PSMA-labelling index = 3 for the evaluated primary tumor and associated cervical lymph node metastasis of this patient. 

## 4. Discussion

PSMA-targeted therapy represents a promising therapeutic option for the treatment of advanced carcinomas and there are currently a number of PSMA-targeted therapeutic approaches being approved for clinical trials e.g., clear cell renal carcinoma, glioblastoma and hepatocellular carcinoma (ClinicalTrials.gov Identifier: NCT02607553, NCT02067156, NCT01777594, NCT01856933) [25]. PSMA-radioligands are, meanwhile, an established theranostic approach with excellent results and less severe side effects for the management of prostate cancer as shown in the VISION study (ClinicalTrials.gov Identifier: NCT03511664) [17,18,26,27]. In contrast, data on the relevance of PSMA-targeted diagnostic and therapy for ATC and PDTC are still lacking. Hence, this study evaluates, to the best of our knowledge, the largest collective of PDTC and ATC patients with advanced metastatic disease investigated by ^68^Ga-PMSA-PET/CT, in terms of theranostics regarding an individual treatment concept in purpose of a ^177^Lu-PSMA therapy. In addition, this study provides the first comparison between IHC and ^68^Ga-PSMA-PET/CT for thyroid cancer patients.

In our cohort, the overall detection rate for PDTC/ATC lesions was 89/91% for the ^18^F-FDG-PET/CT and 11/0% for ^68^Ga-PMSA-PET/CT and thus much lower for the ^68^Ga-PMSA-PET/CT than in prior reports. Verma et al. evaluated ten patients with metastatic differentiated thyroid cancer and found a detection rate of 93.8% for ^68^Ga-PMSA-PET/CT and 81.8% for ^18^F-FDG-PET/CT, while Lawhn-Heath et al. analyzed four patients with dedifferentiated thyroid cancers and reported detection rates of 100% for ^18^F-FDG-PET/CT and 72.7% for ^68^Ga-PMSA-PET/CT [28,29]. One possible explanation for the lower detection rates may be the small patient group collective analyzed in all other studies, which makes differences statistically distinctly divergent. Despite the fact that this study confirms the high diagnostic sensitivity of ^18^F-FDG-PET/CT, it must be further investigated to what extent ^68^Ga-PMSA-PET/CT may offer lower detection rates for dedifferentiated than for differentiated thyroid carcinomas.

However, comparable with preliminary work of other groups, our results showed heterogeneous PSMA expression of the evaluated lesions after ^68^Ga-PMSA-PET/CT imaging—both among patients and lesions [28,29,30,31]. 

According to our results, especially lymph node and bone metastases of dedifferentiated thyroid carcinoma can be visualized in ^68^Ga-PSMA-PET/CT. The varying degree of PSMA expression in the metastases can be caused by different factors, of which neovascularization is of particular importance. The angiogenesis of thyroid carcinoma is controlled by multiple receptors, signaling pathways and genetic targets, which may vary between the primary tumor and different metastases [32]. Furthermore, it is known that the morphology of the tumor angiogenesis influences the tumor environment and can be inhomogeneous between different metastatic sides [33]. Considering these aspects of neovascularization and angioneogenesis, the spectrum of PSMA expression in the investigated metastases, which is mainly found in the tumor-associated neovasculature, can be partly explained.

This study confirmed the previous findings [28,31] highlighting that the lesion FDG uptake was higher than the PSMA uptake in 7/8 evaluated patients with an overall median lesion SUVmax of 5.0 for ^18^F-FDG-PET/CT and 1.5 for ^68^Ga-PMSA-PET/CT in ATC and 17.9 for ^18^F-FDG-PET/CT and 4.0 for ^68^Ga-PMSA-PET/CT in PDTC. Nevertheless, ^68^Ga-PMSA-PET/CT was able to detect metastatic lesions in PDTC including two certain osseous metastases (and two suspicious osseous lesions), which could not be detected by ^18^F-FDG-PET/CT imaging (patient 8), as well as one mediastinal lymph node metastasis and one cerebral metastasis (patient 7). Similar to our findings in patient 7 and 8, Lütje et al. evaluated six patients with iodine-negative metastatic differentiated thyroid carcinoma and found FDG-negative and PSMA-positive metastases in one patient, so we must emphasize at this point that we can expect to identify more of such patients in larger patient collectives, who may benefit from ^68^Ga-PMSA-PET/CT in terms of staging [31].

For a reasonable implementation of individual treatment concepts, all lesions should have a high expression in order to be therapeutically targetable. In one PDTC patient (patient 8), all lesions showed a high PSMA expression; subsequently, as the disease progressed, he received two cycles of ^177^Lu-PSMA therapy, from which he benefited for several months without experiencing serious side effects. Comparing this result with the results of de Vries et al. [30], who showed a slight, temporary response in one of two ^177^Lu-PSMA treated patients and considering the lack of therapeutic options in these cases, PSMA expression offers a promising therapeutic target for a few individually selected PDTC and ATC patients who need alternative treatment options in case of progression when established therapies like tyrosinekinaseinhibitors are no longer effective. Nevertheless, it is important to consider that PSMA in thyroid carcinomas has been detected only in the tumor-associated neovasculature [23]. To achieve a therapeutic effect, the compounds need to the stored in/on/near the tumor cells, due to the short range of the emission (1.5 mm for ^177^Lu). We were able to show visually intense nuclide accumulation in an early scan after 90 min and also in a late scan after at least 43 h of radioligand injection (patient 8), indicating that the neovasculature represents a sufficient target due to a long retention time.

Apart from its well known strong expression in prostate cancer cells, immunohistochemical examination of different solid cancer types, such as clear cell renal carcinoma or lung cancer, demonstrated, in addition to a substantial immunohistochemical PSMA expression, a high tracer accumulation after ^68^Ga-PMSA-PET/CT imaging [34,35,36,37,38,39,40]. Heitkötter et al. analysed 101 thyroid lesions and found a significantly higher PSMA expression in PDTC and ATC tissue sections than in differentiated thyroid tumors [24]. It is therefore not surprising that Sollini et al. demonstrated that PSMA expression in differentiated thyroid carcinomas is associated with more aggressive behavior; therefore, higher expression would have to be expected in dedifferentiated carcinomas [41]. This aspect also fits to the pathogenesis of PSMA expression. It is assumed that PSMA is expressed as part of the autoregulation in tumorigenesis and facilitates endothelial cell invasion of angiogenesis [23]. 

Consistent with the findings from Heitkötter et al. [23], in this study, a strong PSMA expression was identified in the tumor-associated neovasculature of 27 out of 39 ATC and in 13 out of 22 PDTC tissue samples. One PDTC tumor sample even showed a strong PSMA expression of the tumor cells themselves. Furthermore, there was a correlation between immunohistochemical PSMA expression and PSMA uptake on ^68^Ga-PMSA-PET/CT in three of the examined patients (patient 3 and 6 showed a low PSMA expression correspondingly with no PSMA uptake on PET/CT, patient 7 presented a strong PSMA expression correspondingly with an increased PSMA uptake on ^68^Ga-PMSA-PET/CT). On the other hand, four patients enrolled in this study had a high PSMA expression in their tissue samples but no PSMA uptake on the PET/CT examination (patient 1, 2, 4 and 5). A clear explanation for the discrepancy between immunohistochemical PSMA expression in the evaluated tissue samples and uptake on ^68^Ga-PSMA-PET/CT cannot be concluded from the currently available literature and represents, therefore, a topic for further investigations. With regard to prostate carcinoma, it is well known that there is a close correlation between tracer uptake on ^68^Ga-PSMA-PET/CT and PSMA expression in IHC [42]. In contrast, a case study of a glioblastoma was recently published in which a low tracer accumulation was seen in ^68^Ga-PSMA-PET/CT despite an intermediate PSMA expression in IHC [43]. One possible reason for this discrepancy could be that PSMA expression in apical membranes—typical for prostate carcinoma cells—is connected with a higher tracer uptake on PET imaging than the level of PSMA expression in cytoplasm, which is often observed in nonprostatic disease [44]. 

A relevant limitation of this study is the retrospective design and the small patient population due to the rarity of the analyzed carcinoma entities. In addition, the long time interval between ^18^F-FDG- and ^68^Ga-PSMA-PET/CT in some patients might bias our results. Nevertheless we can confirm the high diagnostic sensitivity and superiority of ^18^F-FDG-PET/CT in comparison to ^68^Ga-PSMA-PET/CT in the diagnosis of ATC and PDTC.

It is known from preliminary work of other research groups that both anaplastic and poorly differentiated thyroid carcinoma express GLUT1 (Glucose transporter 1) and that the associated high ^18^F-FDG uptake reflects dedifferentiation of thyroid tumors. Therefore, radioiodine-negative metastases display increased glucose uptake more frequently than radioiodine-positive neoplasms. Furthermore, the up regulation of GLUTs in thyroid cancer seems to be associated with tumor aggressiveness and loss of differentiation [45,46,47]. As a proposal for a consecutive study, the immunohistochemical expression of GLUT1 and associated uptake on ^18^F-FDG-PET/CT could be evaluated and compared to the PSMA expression and imaging in ^68^Ga-PSMA-PET/CT in ATC and PDTC.

Larger prospective studies are urgently needed to conclusively evaluate the impact of PSMA-based diagnosis and therapy for patients suffering from ATC and PDTC.

## 5. Conclusions

In summary, considering the rarity of ATC and PDTC, however, the findings of this study confirm the high diagnostic sensitivity and superiority of ^18^F-FDG-PET/CT in comparison to ^68^Ga-PSMA-PET/CT in the diagnosis of ATC and PDTC. However, it can be suggested that ^68^Ga-PMSA-PET/CT can be considered as a beneficial adjunct to the well-established ^18^F-FDG-PET/CT for a few individual selected patients with advanced and metastatic dedifferentiated and anaplastic thyroid carcinomas to detect lesions not discovered by ^18^F-FDG-PET/CT. In addition, PSMA-targeted therapy can be used as an alternative option in selected patients if they show progression under established therapeutic concepts. Current therapeutic options for ATC and PDTC are limited, so there is a desperate need for novel innovative and targeted treatment modalities. Even though there is a need for larger collectives to allow a final evaluation, radiolabelled PSMA-ligands with ^177^Lu-PSMA may represent in future a theranostic approach with only minor side effects for a rare proportion of these patients.

## Figures and Tables

**Table 1:** Patients´ characteristics at initial diagnosis.

Patient	Sex	Age at Diagnosis (Years)	Year of Diagnosis	Tumor-Type	TNM Stage ^1^	UICC Stage ^1^	Treatment History
1	M	59	2020	ATC	pT4a pN0 M0	IVB	Surgery of the thyroid gland (2020) Radiochemotherapy (2020) Targeted therapy (Pembrolizumab + Lenvatinib) (2020/2021)
2	M	47	2020	ATC	pT4a pN1 M1	IVC	Surgery of the thyroid gland (2020) Radiochemotherapy (2020) Targeted therapy (Pembrolizumab + Lenvatinib) (2020/2021)
3	F	44	2007	PDTC	pT3a pN1 M0	II	Two-stage surgery of the thyroid gland (2007) Radioiodine therapy (3,7 GBq I-131) (2007) Surgery of mediastinal lymph node metastases (2007) Three further radioiodine therapies (cumulative 16,9 GBq I-131) (last 2009) Twofold resection of lung metastases (2012) Fifth radioiodine therapy (5,5 GBq I-131) (2012) mKI therapy (Lenvatinib) (2019)
4	M	51	2015	PDTC	pT4a pN1 M0	II	Two-stage surgery of the thyroid gland (2015) Radioiodine therapy (3,6 GBq I-131) (2015) Surgery vertebral metastasis (2016) Radioiodine therapy (7,4 GBq I-131) (2017) mKI therapy (Lenvatinib) (2018)
5	M	65	2014	PDTC	pT0 cN1 M1	IVB	Surgery of the thyroid gland (oncocytic adenoma) (2010) Surgery of vertebral metastasis (2014) Residual thyroidectomy without tumor detection (2014) Radioiodine therapy (7,4 GBq I-131) (2015) mKI therapy (Sorafenib) (2015) mKI therapy (Lenvatinib) (2015) External beam radiation of osseous metastases (ribs, os ilium, femur right) (2015) Surgery of vertebral metastasis (2017) mKI therapy (Pazopanib) (2017) mKI therapy (Lenvatinib) (2017) COSMIC study inclusion ^2^ (2020)
6	M	70	2010	PDTC	pT2 pN0 M0	I	Two-stage surgery of the thyroid gland (2010) Three cycles of radioiodine therapy (cumulative 14,4 GBq I-131) (last 2011) mKI therapy (Sorafenib) (2015) mKI therapy (Lenvatinib) (2016) mKI therapy (Sorafenib) (2017) mKI therapy (Pazopanib) (2017)
7	F	61	2013	PDTC	pT3a pN1 M1	IVB	Surgery of the thyroid gland (2013) Twofold radioiodine therapy (cumulative 9,9 GBq I-131) (2013) Surgery of cervical lymph node metastasis (2014) Lutetium-177-DOTATATE therapy (2014) mKI therapy (Sorafenib) (2014) External beam therapy of vertebral and cervical lymph node metastases (2016) mKI therapy (Lenvatinib) (2016) Surgery of brain metastases (2018)
8	M	59	2015	PDTC	pT0 cN0 M1	IVB	Surgery of the thyroid gland (2011) Surgery of vertebral metastasis (2015) Residual thyroidectomy without tumor detection (2015) Radioiodine therapy (14,9 GBq I-131) (2015) Two cycles of Lutetium-177-PSMA therapy (cumulativ 13,7 GBq) (2017) mKI therapy (Lenvatinib) (2018) COSMIC study ^2^ inclusion (2020)

^1^ According to the 8th Edition of UICC/TNM Classification ^2^ COSMIC study: Cabozantinib versus placebo in second line therapy of radioiodine refractory thyroid cancer. Abbreviations: M = male, F = female, mKI = multikinaseinhibitor.

**Table 2:** Lesion SUVmax in ^18^FDG-PET/CT and ^68^Ga-PSMA-PET/CT of (metastatic) disease locations and extent of PSMA expression in tissue samples for each patient.

Patient	^18^FDG-PET/CT and ^68^Ga-PSMA-PET/CT Results						PSMA Expression	
	Diagnosis of Primary Tumor	Year of Scan	Interval between ^18^FDG-PET/CT and ^68^Ga-PSMA- PET/CT (Days)	Tumor/Metastases Locations Number of Lesions (*N*) Median Size, cm (Range)	FDG- PET/CT Median SUVmax (Range)	PSMA- PET/CT Median SUVmax (Range)	Tumor Samples	Extend of PSMA- Expression
1	2020	PSMA: 2020 FDG: 2020	1	TB ^1^ (local recurrence) (*n* = 1) 1.9	16.3	2.5	Primary tumor Soft tissue surrounding thyroid gland	Strong expression Strong expression
				LN^2^ (mediastinal) (*n* = 1) 2.2	2.7	3.3		
				PM ^3^ (*n* = 7) 0.7 (0.3–1.2)	2.7 (1.5–7.9)	1.3 (0.6–2.6)		
2	2020	PSMA: 2020 FDG: 2020	1	PM ^3^ (*n* = 2) 2.1; 2.2	21.2 (14.1; 28.3)	3.25 (4.0; 2.5)	Primary tumor Pulmonary metastasis	Strong expression Strong expression
3	2007	PSMA: 2017 FDG: 2016	83	PM ^3^ (*n* > 10) 1.0 (0.5–1.1)	12.6 (6.6–25.3)	3.4 (2.0–4.3)	Primary tumor Lymph node metastasis (cervical) Pulmonary metastasis	Low expression Low expression Low expression
4	2015	PSMA: 2017 FDG: 2016	232	OM ^4^ (vertebra) (*n* = 1) 1.1	7.4	Not per-formed before surgery	Osseous metastases of the vertebra	Strong expression
				LN^2^ (mediastinal) (*n* = 1) 0.8	5.6	2.1		
5	2014	PSMA: 2017 FDG: 2017	40	OM ^4^ (vertebra) (*n* = 1) 4.2	17.9	3.7	Osseous metastases of the vertebra	Strong expression
				OM ^4^ (*n* > 10) 4.2 (2.7–6.1)	17.9 (9.9–31.2)	3.7 (1.8–4.2)		
				TB ^1^ (local recurrence) (*n* = 1) 10.7	19.1	2.4		
6	2010	PSMA: 2017 FDG: 2017	171	TB^1^ (tumor recurrence) (*n* = 1) 10.7	67.9	4.2	Tumor recurrence Lymph node metastasis (cervical)	Low expression Low expression
				LN ^2^ cervical (*n* = 1) 1.9 mediastinal (*n* = 1) 6.7	48.4 55.9	4.1 5.7		
				PM ^3^ (*n* > 10) 1.9 (1.6–3.6)	35.4 (29.4–51.5)	3.8 (2.5–6.3)		
7	2013	PSMA: 2017 FDG: 2016	76	CM ^5^ (*n* = 1) 2.9	31.4	12.3	Primary tumor Lymph node metastasis (cervical)	Strong expression Strong expression
				LN ^2^ cervical (*n* = 1) 2.0 mediastinal (*n* = 2) (3.6; 1.4) inguinal (*n* = 1) 5.4	32.1 30.85 (36.4; 25.3) 35.2	7.4 8.2 (6.3; 10.1) 6.2		
				PM ^3^ (*n* > 10) 1.1 (1.0–1.8)	12.5 (11.7–14.1)	3.3 (2.1–4.0)		
				ST ^6^ (thoracic wall) (*n* = 1) 2.1	31.0	4.3		
				OM ^4^ (Os occipital) (*n* = 1) 2.9	16.9	7.2		
8	2015	PSMA: 2016 FDG: 2016	81	OM ^4^ (*n* = 4) 1.25 (0 *–1.6)	2.3 (1.2–2.7)	5.55 (2.9–9.0)	No tissue sample of this patient was available	

^1^ TB = thyroid bed, ^2^ LN = lymph nodes, ^3^ PM = pulmonary/pleural metastases, ^4^ OM = osseous metastases, ^5^ CM = cerebral metastases, ^6^ ST = soft tissue * FDG and PSMA uptake in this lesion without a morphological correlate.

**Table 3:** Median lesions SUVmax and discordant imaging of metastatic sides of ATC and PDTC on ^68^Ga-PSMA- and ^18^F-FDG-PET/CT.

Disease Location	^68^Ga-PSMA- PET/CT Median SUVmax (Range) *	Clearly (Discreet) PSMA-Positive Lesions/ Total Rated Lesions	^18^FDG-PET/CT Median SUVmax (Range) *	Clearly (Discreet) FDG-Positive Lesions/ Total Rated Lesions ^1^	Number of Clearly Dis-Cordant Lesions ^2^/Total Rated Lesions ^1^	Description of Clearly Discordant Lesions ^1^
**ATC**						
Mediastinal lymph node	3.3	0 (1)/1	2.7	0 (1)/1	0/1	-
Pulmonary metastases	1.3 (0.6–4)	0 (0)/9	5.0 (1.5–28.3)	9 (0)/9	9/9	9 FDG+, PSMA−
Local tumor recurrence/ thyroid bed lesions	2.5	0 (0)/1	16.3	1 (0)/1	1/1	1 FDG+, PSMA−
**PDTC**						
Osseous metastases	3.85 (1.8–9.0)	2 (3)/10	9.9 (1.2–31.2)	6 (0)/10	7/10 ^3^	2 PSMA+, FDG− 5 FDG+, PSMA-
Cervical lymph nodes	5.75 (4.1–7.4)	0 (1)/2	40.25 (32.1–48.4)	2 (0)/2	1/2 ^4^	1 FDG+, PSMA−
Mediastinal lymph nodes	6.0 (2.1–10.1)	1 (1)/4	30.85 (5.6–55.9)	4 (0)/4	2/4 ^5^	2 FDG+, PSMA−
Pulmonary metastases	3.45 (2.0–6.3)	0 (0)/15	15.5 (6.6–51.5)	15 (0)/15	15/15	15 FDG+, PSMA−
Local tumor recurrence/thyroid bed lesions	3.3 (2.4–4.2)	0 (0)/2	43.5 (19.1–67.9)	2 (0)/2	2/2	2 FDG+, PSMA−
Cerebral metastases	12.3	1 (0)/1	31.4	1 (0)/1	0/1	-
Soft tissue/others (inguinal lymph node metastasis)	5.25 (4.3–6.2)	0 (1)/2	33.1 (31–35.2)	2 (0)/2	1/2 ^4^	1 FDG+, PSMA−

* If only one lesion of a disease location is present, only the SUVmax for the respective lesion is given. ^1^ The patient with surgery of known FDG-positive vertebral metastases after ^18^FDG-PET/CT was excluded for absence of comparability. ^2^ Only the clearly discordant lesions are described here. Lesions that had a discrete and thus not clearly positive uptake were not evaluated. ^3^ Of ten lesions, three are discretely PSMA- and FDG-positive, which is why they are not evaluated as clearly discordant. ^4^ Of two lesions, one is discretely PSMA- and FDG-positive, which is why it is not evaluated as clearly discordant. ^5^ Of four lesions, one is PSMA- and FDG-positive (concordant); one is discretely PSMA- and FDG-positive, which is why it is not evaluated as clearly discordant. + = positive; − = negative.

**Table 4:** Correlation between the extent of immunohistochemical PSMA expression and imaging in ^68^Ga-PSMA-PET/CT with regard to different metastatic sides of ATC and PDTC patients.

Immunohistochemical Analysis of ATC and PDTC Tissue Samples ^1^					Imaging Analysis ^2^	
Metastatic Side	No PSMA Expression	PSMA-Labelling Index 1 “Low Expression”	PSMA-Labelling Index 2 “Strong Expression”	PSMA-Labelling Index 3 “Strong Expresion”	Clearly PSMA-Positive Lesions/Total Rated Lesions	Discreet PSMA-Positive Lesions/Total Rated Lesions
**ATC**						
Pulmonary/pleural metastases (*n* = 1)	0	0	1	0	0/2	0/2
**PDTC**						
Cervical lymph node metastases (*n* = 2)	0	1	1	0	0/2	1/2
Pulmonary metastases (*n* = 1)	0	1	0	0	0/>10	0/>10
Local recurrence (*n* = 1)	0	1	0	0	0/1	0/1
Osseous metastases (*n* = 1)	0	0	1	0	0/>10	0/>10

^1^ PSMA-labelling index 1, “low expression”: weak or moderate staining intensity in <5% of the neovasculature.—weak staining intensity in >5% of the neovasculature. PSMA-labelling index 2, “strong expression”: moderate staining intensity in >5% of the neovasculature.—strong staining intensity < or >5% of the neovasculature. PSMA-labelling index 3, “strong expression”: strong staining intensity not only in the neovasculature, but also in the tumor cells. ^2^ For explanation refer to Table 3.

**Table 5:** **IHC analysis:** Extend of PSMA expression in tissue samples of ATC and PDTC.

Disease Location	No of Total Tissue Samples	Tissue Samples without Any PSMA Expression	Tissue Samples with “Low Expression“ ^1^, PSMA-Labelling Index 1	Tissue Samples with “Strong Expression“ ^2^, PSMA-Labelling Index 2	Tissue Samples with “Strong Expression” ^2^, PSMA-Labelling Index 3
**ATC**					
Primary tumor	22	1	3	18	0
Lymph node metastases	5	1	1	3	0
Pulmonary/pleural metastases	4	0	1	3	0
Soft tissue—surrounding thyroid gland—presternal metastasis	7 1	1 0	3 1	3 0	0 0
**PDTC**					
Primary tumor	6	0	1	4	1
Lymph node metastases	6	0	3	2	1
Pulmonary metastases	3	0	1	2	0
Others—renal metastasis—local recurrence	1 2	0 0	0 1	1 1	0 0
Osseous metastases	4	1	0	3	0

^1^ “low expression”:—weak or moderate staining intensity in <5% of the neovasculature.—weak staining intensity in >5% of the neovasculature. ^2^ “strong expression”:—moderate staining intensity in >5% of the neovasculature.—strong staining intensity < or >5% of the neovasculature.—strong (3) staining intensity not only in the neovasculature, but also in the tumor cells.

## Data Availability

The data presented in this study are available upon request from the corresponding author. The data are not publicly available due to ethical restrictions.

## Supplementary Materials

The following are available online at https://www.mdpi.com/article/10.3390/cancers13225688/s1, Text S1: Patient 2: male, ATC, 47 years old at the time of diagnosis, Text S2: Patient 3: female, PDTC, 44 years old at the time of diagnosis, Text S3: Patient 4: male, PDTC, 51 years old at the time of diagnosis, Text S4: Patient 5: male, PDTC, 65 years old at the time of diagnosis, Text S5: Patient 6: male, PDTC, 70 years old at the time of diagnosis, Figure S1: Patient 2: 68Ga-PSMA-PET/CT (A,B), 18FDG-PET/CT (C,D), Figure S2: Patient 3: 68Ga-PSMA-PET/CT (A,B) 18FDG-PET/CT (C,D), Figure S3: Immunohistochemistry of patient 3: (A) and (B), histology of PDTC thyroidal primary tumor, (C) and (D) lymph node metastasis and (E) and (F), lung metastasis in HE staining and PSMA immunohistochemistry, respectively, Figure S4: Patient 4: 68Ga-PSMA-PET/CT (A,B), 18FDG-PET/CT (C,D), Figure S5: Patient 5: 68Ga-PSMA-PET/CT (A,B), 18FDG-PET/CT (C,D), Figure S6: Patient 6: 18FDG-PET/CT (A,B), 68Ga-PSMA-PET/CT (C,D).
