# Supplementary material for: Prostate-Specific Membrane Antigen in Anaplastic and Poorly Differentiated Thyroid Cancer—A New Diagnostic and Therapeutic Target?
Source: Cancers (Basel). 2021 Nov 14;13(22):5688. doi: 10.3390/cancers13225688 (PMC8616254; doi:10.3390/cancers13225688)
Supplement: Supplementary file 1 [file cancers-13-05688-s001.zip › cancers-1443233-supplementary.pdf]

Supplementary Material: Prostate-Specific Membrane Antigen in Anaplastic and Poorly Differentiated Thyroid Cancer—A New Diagnostic and Therapeutic Target?

Sabine Wächter, Pietro Di Fazio, Elisabeth Maurer, Jerena Manoharan, Corinna Keber, Andreas Pfestroff, Damiano Librizzi, Detlef K. Bartsch, Markus Luster and Friederike Eilsberger

Text S1. Patient 2: male, ATC, 47 years old at the time of diagnosis

As part of the follow-up after performing thyroid surgery, as well as radiochemotherapy, a ^{18}FDG-PET/CT and ^{68}Ga-PMSA-PET/CT were performed eight months after initial diagnosis. The time interval between these two examinations was one day.

The ^{18}FDG-PET/CT showed two pulmonal lesions, one in the right upper lobe pleural of 2.2 cm with increased glucose uptake (SUVmax 28.3) without increased PSMA expression (SUVmax 2.5) and one in the right lower lobe of 2.1 cm with increased glucose uptake (SUVmax 14.1) likewise without increased PSMA expression (SUVmax 4.0) (Figure S1).

The primary tumor as well as a pulmonary metastasis were examined with regard to their PSMA expression and revealed a "strong expression" (PSMA-labelling index = 2).

In summary, this patient showed two metastases with increased glucose metabolism without PSMA expression in PET/CT, despite a strong PSMA expression in the examined tissue samples.

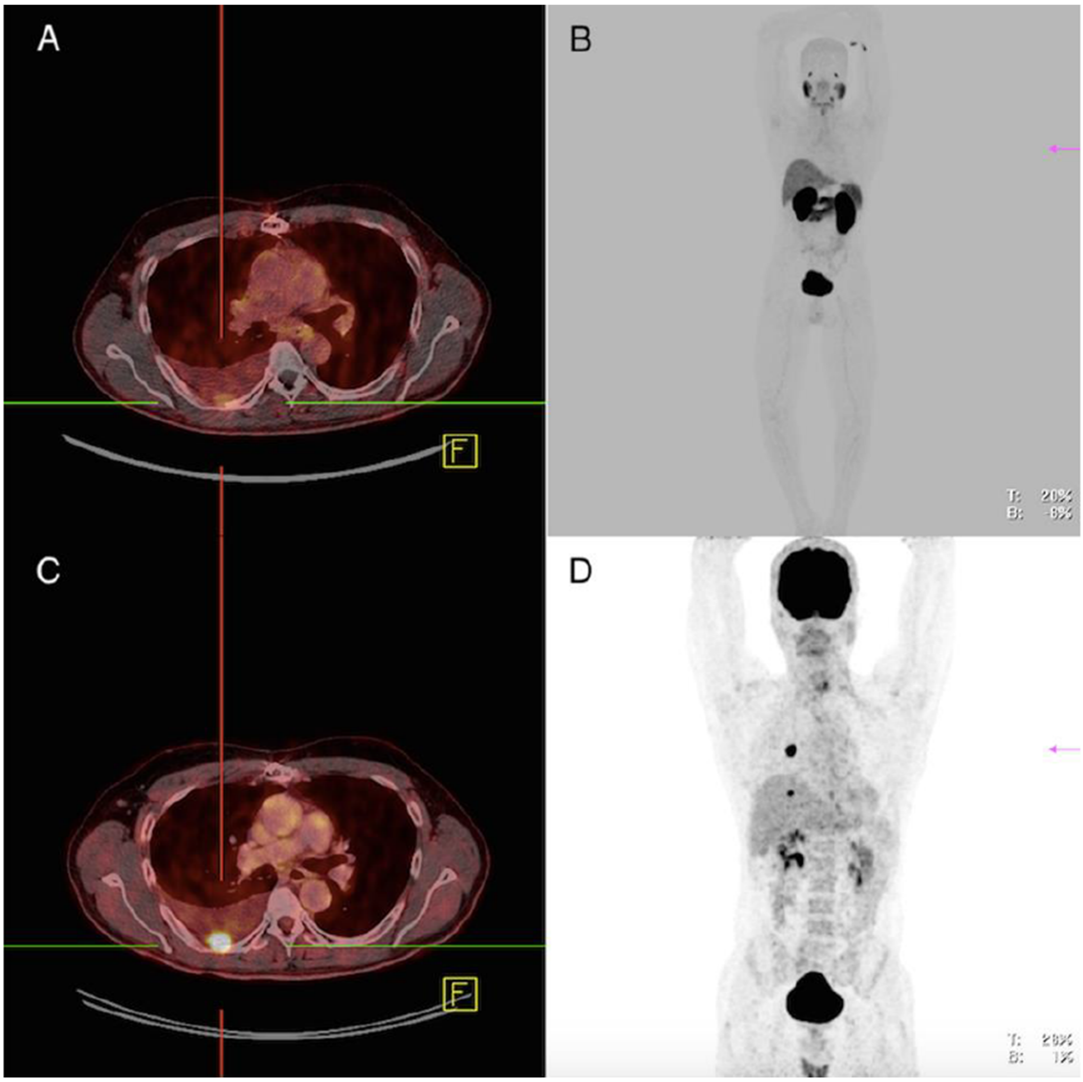

Figure S1. Patient 2: ^{68}Ga-PSMA-PET/CT (**A,B**), ^{18}FDG-PET/CT (**C,D**). The finding was consistent with pleural/pulmonary metastasis with increased FDG tracer accumulation, without PSMA expression.

Text S2. Patient 3: female, PDTC, 44 years old at the time of diagnosis

After two-stage surgery of the thyroid gland and mediastinal lymph node metastases in 2007, as well as five radioiodine therapies between 2007 and 2012 and twofold resection of lung metastases in 2012 a ^{18}FDG-PET/CT was performed as part of the follow-up 112 months after initial diagnosis. ^{68}Ga-PMSA-PET/CT was performed 83 days later. There was no change in therapy between these examinations. ^{18}FDG-PET/CT showed more than ten pulmonary metastases, so we describe only the five most conclusive ones. On the right side of segment 2, a 0.7 cm solid lesion presented with increased glucose utilization (SUVmax 16.9) without increased PSMA expression (SUVmax 2.0). A 0.5 cm metastasis in segment 3 on the right showed increased FDG uptake (SUVmax 6.6) without increased PSMA expression (SUVmax 2.2), a 1.0 cm lesion with increased glucose uptake (SUVmax 25.3) without PSMA expression (SUVmax 3.4) was also seen in this segment. In the left lung, exemplary lesions presented in segment 8 were a 1.1 cm lesion and in segment 7 a 1.0 cm lesion with increased glucose uptake (SUVmax 12.5 and 12.6) without increased PSMA expression (SUVmax 4.1 and 4.3) (Figure S2).

The primary tumor, a cervical lymph node and a pulmonary metastasis could be histologically examined and presented a low PSMA expression (PSMA-labelling index 1) (Figure S3).

In summary, this patient showed multiple pulmonal metastases with increased glucose metabolism without PSMA expression in PET/CT, compatible with a low PSMA expression in the examined tissue sample.

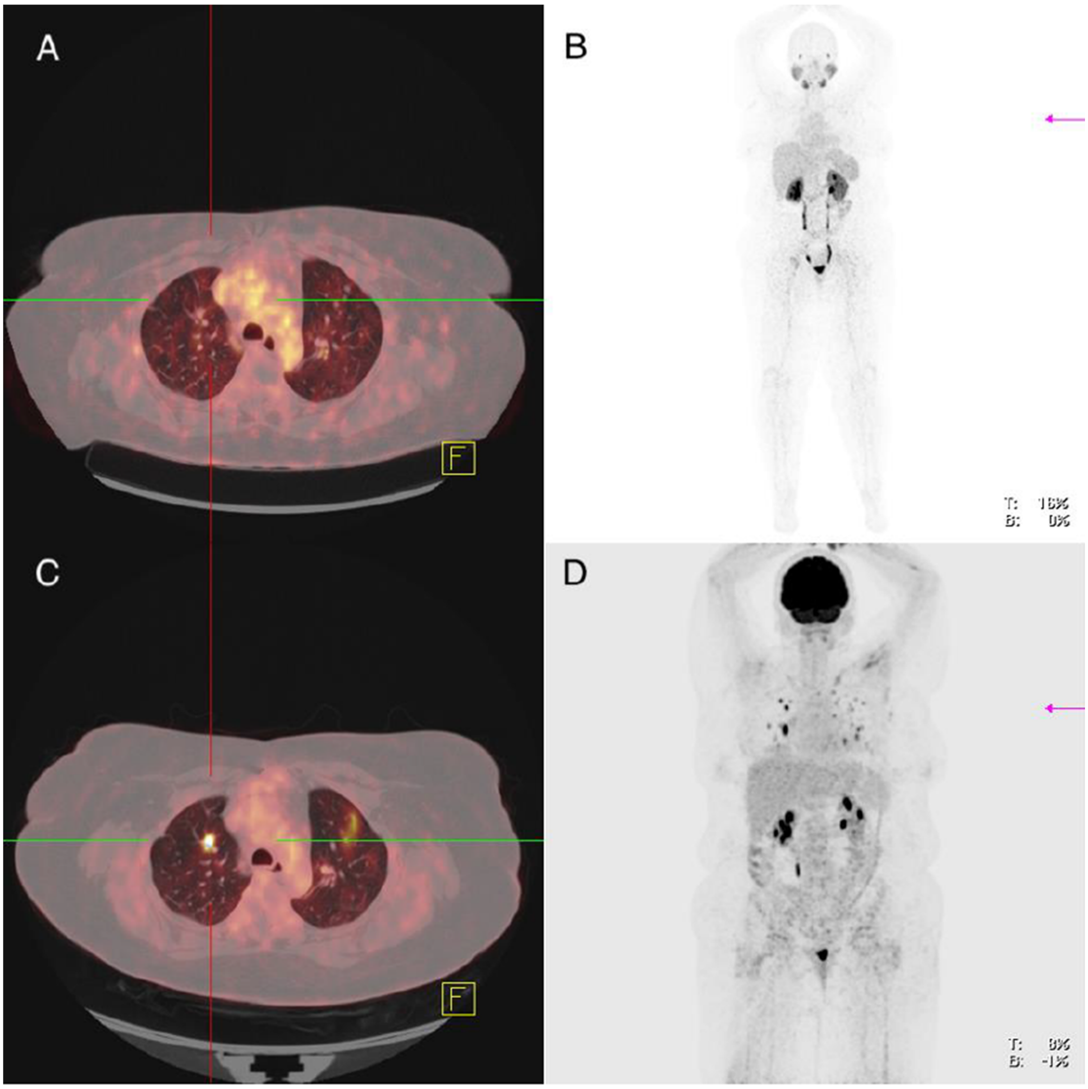

Figure S2. Patient 3: 68Ga-PSMA-PET/CT (**A,B**) 18FDG-PET/CT (**C,D**). FDG-PET/CT showed multiple lung metastases with increased glucose uptake without increased PSMA expression.

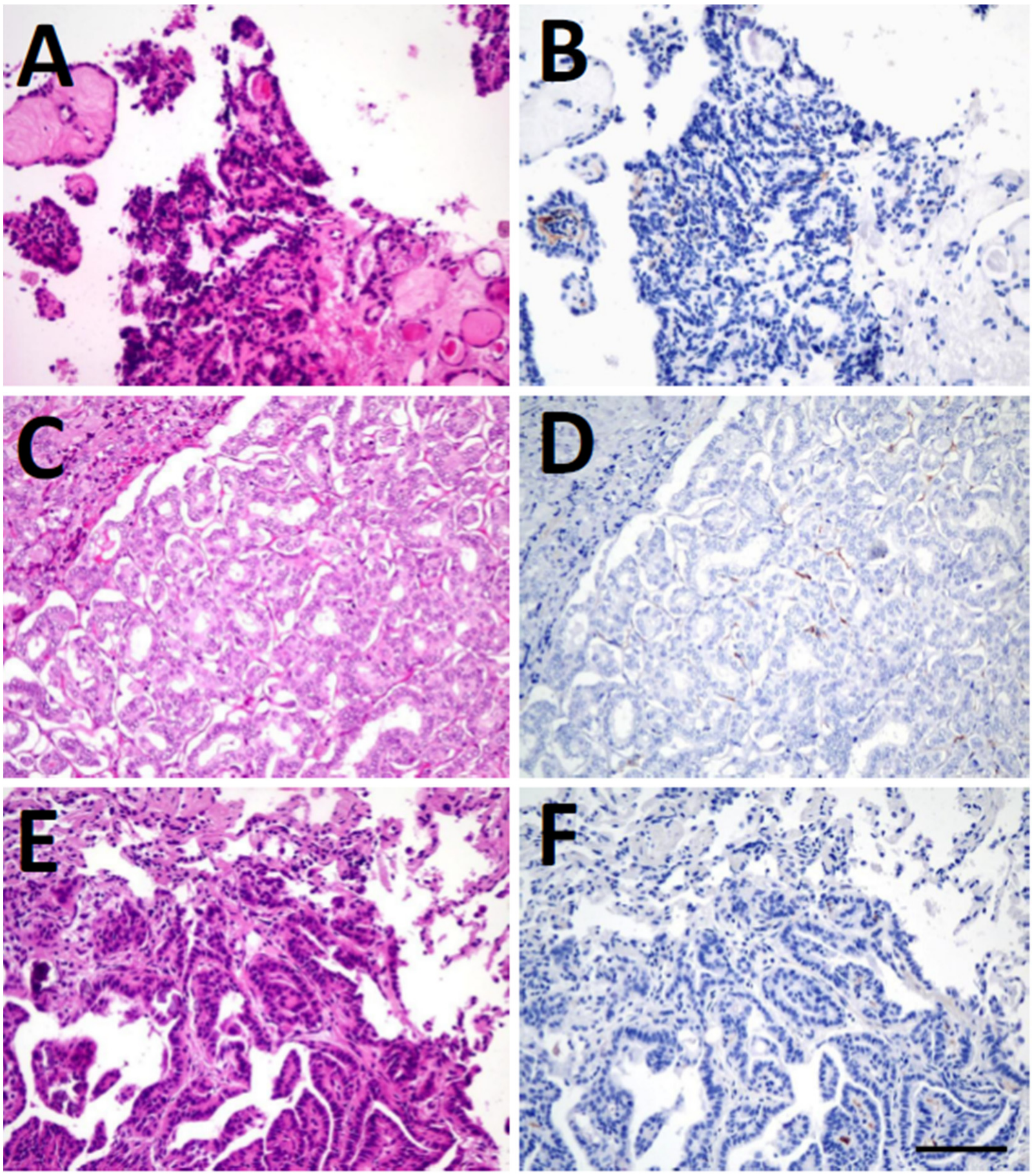

Figure S3. Immunohistochemistry of patient 3: (**A**) and (**B**), histology of PDTC thyroidal primary tumor, (**C**) and (**D**) lymph node metastasis and (**E**) and (**F**), lung metastasis in HE staining and PSMA immunohistochemistry, respectively. Moderate immunoreactivity of less than 5% of the neovasculature in the tumor can be seen at all three localizations (PSMA-labelling index 1). Scale bar, 100 μM, respectively.

Text S3. Patient 4: male, PDTC, 51 years old at the time of diagnosis.

As part of the follow-up after thyroidectomy, as well as radioiodine therapy, a 18FDG-PET/CT was performed 15 months after initial diagnosis and 68Ga-PMSA-PET/CT was performed 232 days later. Between the 18FDG-PET/CT and the 68Ga-PMSA-PET/CT the patient underwent surgery for the known spinal metastasis, which in this study showed osteolysis of 1.1 cm and increased glucose uptake (SUVmax 7.4). Furthermore, the preoperative contrast agent supported 18FDG-PET/CT revealed a mediastinal lymph node of 0.8 cm with increased glucose uptake (SUVmax 5.6). Since 68Ga-PMSA-PET/CT was performed after surgery, it is not possible to comment on the bone metastasis examined. The previously described mediastinal lymph node could probably not be clearly delineated due to the lack of contrast medium, functionally there was no increased PSMA expression (SUVmax 2.1) (Figure S4).

The pathological examination of the osseous metastasis of the thoracic vertebra revealed a strong PSMA expression (PSMA-labelling index = 2).

In summary, this patient showed a mediastinal lymph node metastasis with increased glucose metabolism without PSMA expression in PET/CT, despite a strong PSMA expression in the examined tissue sample.

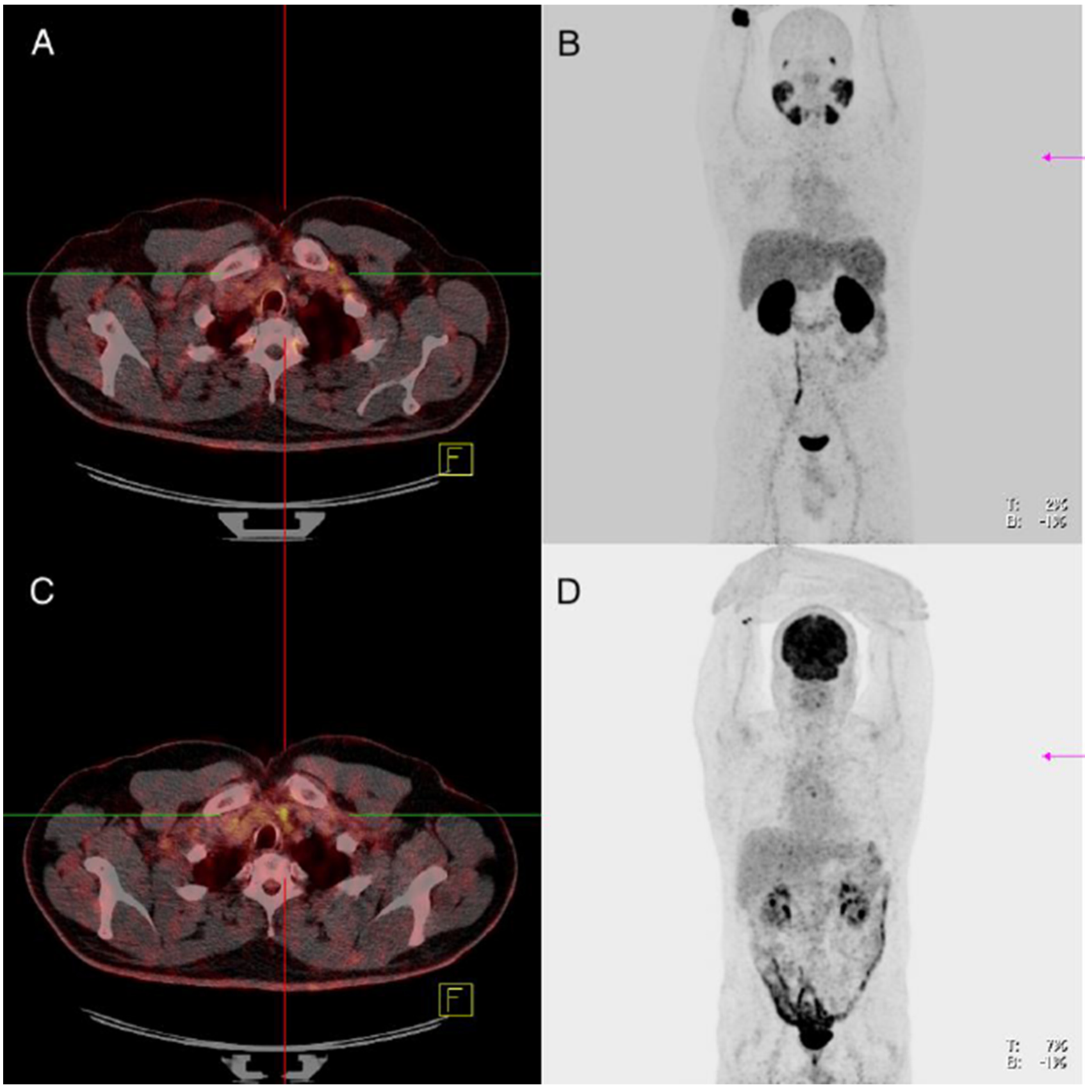

Figure S4. Patient 4: 68Ga-PSMA-PET/CT (**A**,**B**), 18FDG-PET/CT (**C**,**D**). The finding was consistent with a suspected mediastinal lymph node metastasis on FDG imaging, without PSMA expression.

Text S4. Patient 5: male, PDTC, 65 years old at the time of diagnosis.

In this patient 18FDG-PET/CT was performed 26 months after initial diagnosis after receiving thyroidectomy for oncocytic adenoma of the thyroid gland in 2010, resection of a vertebral metastasis in 2014, cervical reexploration with residual thyroidectomy without tumor detection in 2014, radioiodine therapy in 2015 and mKI therapy with sorafenib and lenvatinib in 2015. 68Ga-PMSA-PET/CT was performed 40 days after the 18FDG-PET/CT. There was no change in treatment strategies between the two examinations, however, at the time of the PET/CTs, the patient was in disease progression on ongoing therapy with lenvatinib. 18FDG-PET/CT showed increased glucose uptake of an osseous lesion of the vertebra of 4.2 cm (SUVmax 17.9), whereas 68Ga-PMSA-PET/CT showed no increased PSMA expression of this lesion (SUVmax 3.7) (Figure S5). Furthermore, multiple FDG-positive osseous lesions were shown - for example in the 5th and 7th rib on the right of 6.1 cm and 4.4 cm, os ilium on the right of 3.1 cm and femoral on the right of 2.7 cm (SUVmax 9.9; 31.2; 16.7; 19.3), all showing no increased PSMA expression (SUVmax 3.7; 4.2; 2.5; 1.8). In addition, the left paratracheal region presented with suspected local recurrence

with increased glucose uptake (SUVmax 19.1) without increased PSMA expression (SUVmax 2.4).

In this patient, the material of osseous metastases from thoracic vertebral bodies 8 and 12 could be histopathological examined and revealed a strong PSMA expression (PSMA-labelling index = 2).

In summary, this patient showed multiple osseous metastases with increased glucose metabolism without PSMA expression, despite a strong PSMA expression in the evaluated tissue samples.

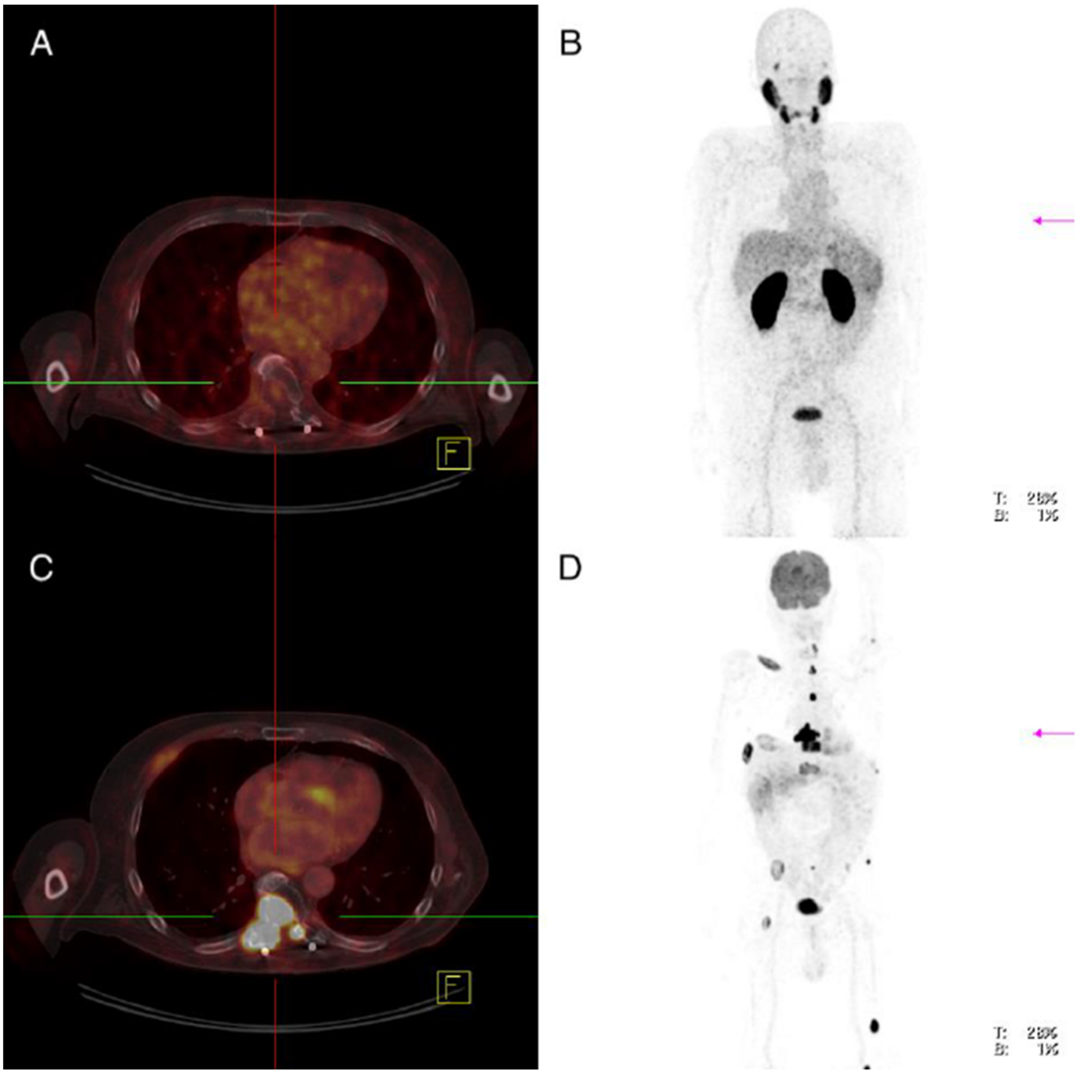

Figure S5. Patient 5: 68Ga-PSMA-PET/CT (**A**,**B**), 18FDG-PET/CT (**C**,**D**). Increased glucose utilization in the histopathological examined spinal metastases without increased PSMA expression.

Text S5. Patient 6: male, PDTC, 70 years old at the time of diagnosis.

After two-stage surgery of the thyroid gland in 2010, the patient received three cycles of radioiodine therapy until 2011 and mKI therapies with sorafenib (2015) and lenvatinib (2016). 82 months after initial diagnosis 68Ga-PSMA-PET/CT was performed in the clear setting of theranostic when therapeutic options were exhausted after these two different mKI therapies and tumor progress. 18FDG-PET/CT was conducted 171 days later. Between the examinations this patient had an interruption of re-challenge with sorafenib for severe unspecified adverse events. 18FDG-PET/CT showed a local recurrence of 10.7 cm in diameter with increased glucose uptake (SUVmax 67.9), adjacent FDG-positive right cervical lymph node metastasis up to 1.9 cm (SUVmax 48.4) and mediastinal lymph node metastasis up to 6.7 cm in regio 6 with glucose uptake (SUVmax 55.9). None of these lesions showed increased PSMA expression (SUVmax 4.2; 4.1; 5.7) Additionally there were

multiple lung metastases bilaterally exemplary in the right lung segment 5 of 1.6 cm, segment 9 of 2.5 cm and in the left lung segment 3 of 1.7 and 1.9 cm and segment 10 of 3.6 cm with an increased FDG-uptake (SUVmax 35.4 (range 29.4-51.5) None of the pulmonary lesion showed increased PSMA expression (SUVmax 3.8 (range 2.5-6.3) (Figure S6).

Samples of tumor recurrence as well as a cervical lymph node metastasis were examined with regard to their PSMA expression and revealed a "low expression" (PSMA-labelling index = 1).

In summary, this patient showed a local recurrence and multiple pulmonal metastases with increased glucose metabolism without PSMA expression in PET/CT, compatible with a low PSMA expression in the examined tissue sample.

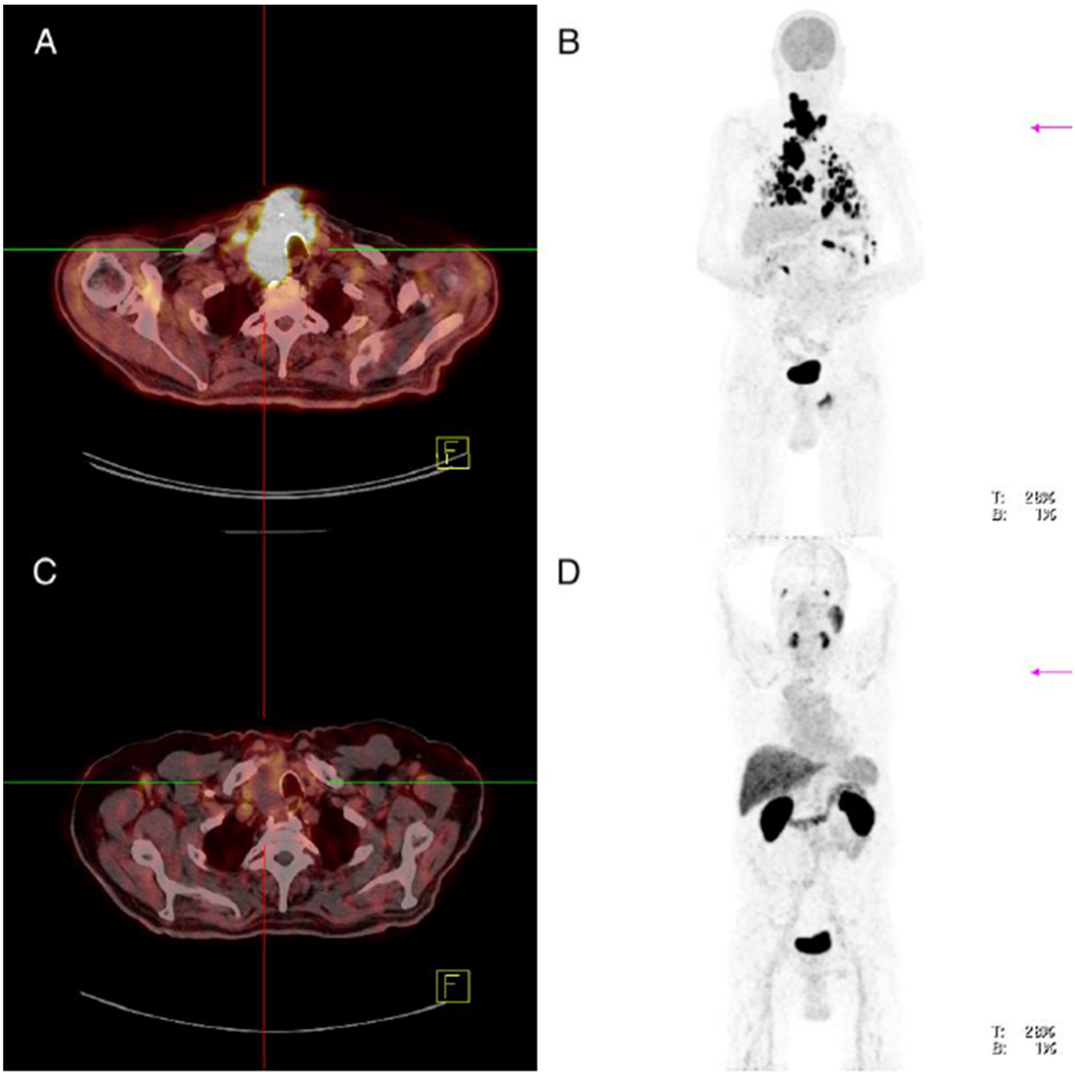

Figure S6. Patient 6: 18FDG-PET/CT (**A**,**B**), 68Ga-PSMA-PET/CT (**C**,**D**). The finding is consistent with tumor recurrence, cervical lymph node metastasis and multiple lung metastases on FDG imaging, without PSMA expression.

Publisher's Note: MDPI stays neutral with regard to jurisdictional claims in published maps and institutional affiliations.
